# DSab-origin: a novel IGHD sensitive VDJ mapping method and its application on antibody response after influenza vaccination

**DOI:** 10.1186/s12859-019-2715-7

**Published:** 2019-03-14

**Authors:** Qingchen Zhang, Lu Zhang, Chen Zhou, Yiyan Yang, Zuojing Yin, Dingfeng Wu, Kailin Tang, Zhiwei Cao

**Affiliations:** Shanghai 10th people’s hospital, School of Life Sciences and Technology, Tongji University, Shanghai, 200092 People’s Republic of China

**Keywords:** Immunoglobulin, V(D)J rearrangements, Influenza infection, Antibodies, Vaccine

## Abstract

**Background:**

Functional antibody genes are often assembled by VDJ recombination and then diversified by somatic hypermutation. Identifying the combination of sourcing germline genes is critical to understand the process of antibody maturation, which may facilitate the diagnostics and rapid generation of human monoclonal antibodies in therapeutics. Despite of successful efforts in V and J fragment assignment, method in D segment tracing remains weak for immunoglobulin heavy diversity (IGHD).

**Results:**

In this paper, we presented a D-sensitive mapping method called DSab-origin with accuracies around 90% in human monoclonal antibody data and average 95.8% in mouse data. Besides, DSab-origin achieved the best performance in holistic prediction of VDJ segments assignment comparing with other methods commonly used in simulation data. After that, an application example was explored on the antibody response based on a time-series antibody sequencing data after influenza vaccination. The result indicated that, despite the personal response among different donors, IGHV3–7 and IGHD4–17 were likely to be dominated gene segments in these three donors.

**Conclusions:**

This work filled in a computational gap in D segment assignment for VDJ germline gene identification in antibody research. And it offered an application example of DSab-origin for studying the antibody maturation process after influenza vaccination.

**Electronic supplementary material:**

The online version of this article (10.1186/s12859-019-2715-7) contains supplementary material, which is available to authorized users.

## Background

Antibody undergoes genetic recombination and somatic hypermutation to achieve the diversity of immune repertoires during the maturation. The diversity of the immunoglobulin is firstly generated by the recombination of variable V, diversity D, and joining J gene segments with imprecise junctions formed by palindromic and non-templated nucleotides [1, 2]. After that, somatic hypermutation creates further diversity by introducing point mutations into the rearranged immunoglobulin variable domain to enhance the affinity between the antibody and antigen [3]. Among the whole process, D segment of antibody heavy chain (IGHD) was found to play a critical role in forming the majority Complementarity Determining Region 3 (CDR3) region binding directly to the epitope of antigens [4–6]. Despite of some progress in the study of antibody maturation, it is still unclarified that how the antigen elicits the antibody maturation and development. Exploration of potential patterns in this process can not only offer important insights into the antibody maturation, but also lead to the future diagnostics and therapeutics [7–9].

Since the VDJ assignment lays a foundation for the research of B cell repertoire, lots of works have been achieved in methodology. Methods for tracing back VDJ gene segments fall into alignment-based methods [10–12], model-based methods [13–15] and others [16]. For instance, Ab-origin was designed on empirical knowledge, optimized scoring scheme and appropriate parameters with aligning query against germline databases [12]. IgBLAST was developed based on the BLAST algorithm [10, 17]. While JOINSOLVER was developed with alignment-based method specifically for analyzing CDR3 regions [18]. In order to model the processes involved in human IGH gene rearrangement and maturation, iHMMune-align took advantages of a hidden Markov model (HMM) [13]. But, due to the VDJ gene recombination, palindromic and non-templated nucleotide additions, and somatic hypermutation implemented during the process of antibody maturation, it is difficult to trace VDJ gene segments back to the germline, especially for D gene segments.

Among the studies of antibody development, seasonal pandemics of Influenza A are frequently used as an example due to the continuous and serious threat to global health. Two major proteins, hemagglutinin (HA) and neuraminidase (NA), locate in the surface of Influenza A, where HA is the main protein that elicits HA-positive neutralizing antibodies. After influenza virus infection or vaccination, antibody-secreting B cells (ASCs) proliferate rapidly and release huge amounts of antibodies, while some other HA-positive B cells differentiate into activated B cells (ABCs). In contrast to ASCs, these ABCs, which are activated without secreting antibodies, are classified as memory B cells (MBCs) lineage [19].

Utilizing next-generation sequencing (NGS) technology, B cell response has been depicted at genomic level after influenza infection or vaccination recently [20–22]. Krause’s work indicated that IGHV3–7/IGHJ6 was used as a dominated gene segments by studying of peripheral blood mononuclear cell (PBMC) sequencing dataset from a 47-year-old healthy woman after the H1N1 pandemic, and suggested that a wide diversity of somatic variants may facilitate recognition in rapidly mutating virus epitopes [23]. Avnir studied a cohort of National Institutes of Health (NIH) H5N1 vaccines, which showed the dominance of F-alleles in HV1–69-sBnAbs on V-segment usage [24]. These works opened the insights of repertoire development, but the samples are rather limited, and IGHD was seldom studied because of the absence of IGHD sensitive mapping method.

Importantly, Ellebedy’s work produced 18 sets of high quality sequencing data of IGH repertoires in time-series of three donors after Trivalent Influenza Vaccine (TIV) vaccination [19]. Although we should note that the datasets is small for a definite conclusion, it offers us the opportunity to give an example for the application of DSab-origin. In this study, we constructed an IGHD sensitive method DSab-origin to improve the D gene assignment of immunoglobulin. Then, our method was applied to analyze the 18 datasets according to time-series of 0, 7, 28, 90 days, which covered naive B cells, MBCs, ABCs and ASCs from three donors [19].

## Results

### DSab-origin algorithm and performance validation

#### DSab-origin algorithm construction

Since the variable region of antibody heavy chain consists of variable V, diversity D, and joining J gene segments with imprecise nucleotide additions adjacent to the D gene segment, the query is artificially divided into three parts: V block (variable V), NDN block (diversity D and additions), and J block (joining J). To separate these three parts, we first identified the germline V and J gene hits with the human IGHV and IGHJ germline repertoires obtained from IMGT [25] via performing BLAST searches [17]. After identified the best matched germline gene hit, we removed the V and J block in the query sequence by aligning with the hit. Then the remaining NDN block was processed by modified k-mers algorithm considering the mutable preference of antibody sequence. The top matched D gene and imprecise nucleotide additions were identified with the scoring strategy.

#### DSab-origin performance on different datasets

Firstly, we validated the performances of DSab-origin on IGHD with unique sequences data. Two standard data sets with 57 and 99 unique sequences, respectively, from tonsillar IgG class-switched B cell were employed to evaluate DSab-origin performance in D gene segment prediction [26]. There were 7 somatic mutations of 31 sites (22.58%) for IGHD3–10*1, and 3 somatic mutations of 18 sites (16.67%) for IGHD6–6*01 in 57 and 99 datasets separately. The accuracies of DSab-origin prediction were 92.3 and 85.3% in identifying the known IGHD gene alleles (IGHD3–10*1 for 57 sequences data set and IGHD6–6*01 for 99 sequences data set), which were the most agreement of four common methods (iHMMune, V-Quest, SoDa and JOINSOLVER) in Gaeta’s work [13]. DSab-origin was also validated on the assignment of mouse D gene segment. The testing datasets were derived from the sequencing of productive preassembled VDJ allele encoding the immunoglobulin heavy chain in mouse [27]. The average accuracy of D gene allele assignments that DSab-origin gave was 95.8% among six test datasets.

In addition, an experimental data with multiple VDJ gene usages was employed to test the overall performance of DSab-origin on IGHV, IGHD and IGHJ segments prediction. S22 Stanford dataset [28] with the real mutability came from an individual who was fully genotyped, but there was an absent of certain VDJ gene segments usage. To overcome this situation, if four or more assignments of five methods (igBLAST [10], IMGT/V-QUEST [11], VDJ [29], VDJalign [14], Cloanalyst [30]) [16] were consistent in one query, it was regarded as reference VDJ gene segments usage. After that, 10,467 sequences were filtered out from altogether 13,153 sequences. DSab-origin returned the correct allele in the set of VDJ gene assignments in 97.45, 97.71 and 99.59%, respectively. To evaluate the performance of DSab-origin, we compared the prediction results with other five common used algorithms. The result indicated that DSab-origin predicted with more than 97% correct alleles in S22 Stanford datasets, while other algorithms had a lower accuracy in IGHV and IGHD prediction (Additional file 1: Table S2).

To evaluate the performance of DSab-origin degrade as somatic hyper-mutation rates increase, we generated 10 to 100% mutation rates with a step of 10% using [31]. The accuracies maintained around 90% as somatic hyper-mutation rates increase (Additional file 2: Figure S1).

### The comparison between DSab-origin and other methods

The performance of DSab-origin was also compared with several commonly used methods. In two standard data sets with 57 and 99 unique sequences [26], DSab-origin gave the highest accuracy comparing with IgBLAST, IMGT/V-QUEST, and iHMMune-align (Table 1). And in above mouse immunoglobulin heavy chain data (LS288–293) [27], DSab-origin and other three methods (igBLAST, IMGT/V-QUEST, iHMMune-align) all achieved high accurate D gene allele assignments (Table 1).

**Table 1:** Comparative method performance on D gene segment

	DSab-origin	iHMMune-align	V-quest	igBlast
57 Sequences (%) [26]	92.3	72.3	12	71.9
99 Sequences (%) [26]	85.3	81.1	83.2	44.2
LS288 (%) [27]	97.01	35.39	96.33	95.55
LS289 (%) [27]	95.11	35.08	94.96	94.26
LS290 (%) [27]	95.89	36.12	94.61	95.94
LS291 (%) [27]	95.53	34.04	93.6	95.14
LS292 (%) [27]	96.86	37.37	94.07	96.13
LS293 (%) [27]	94.4	33.04	91.55	93.04

**Fig. 1:**
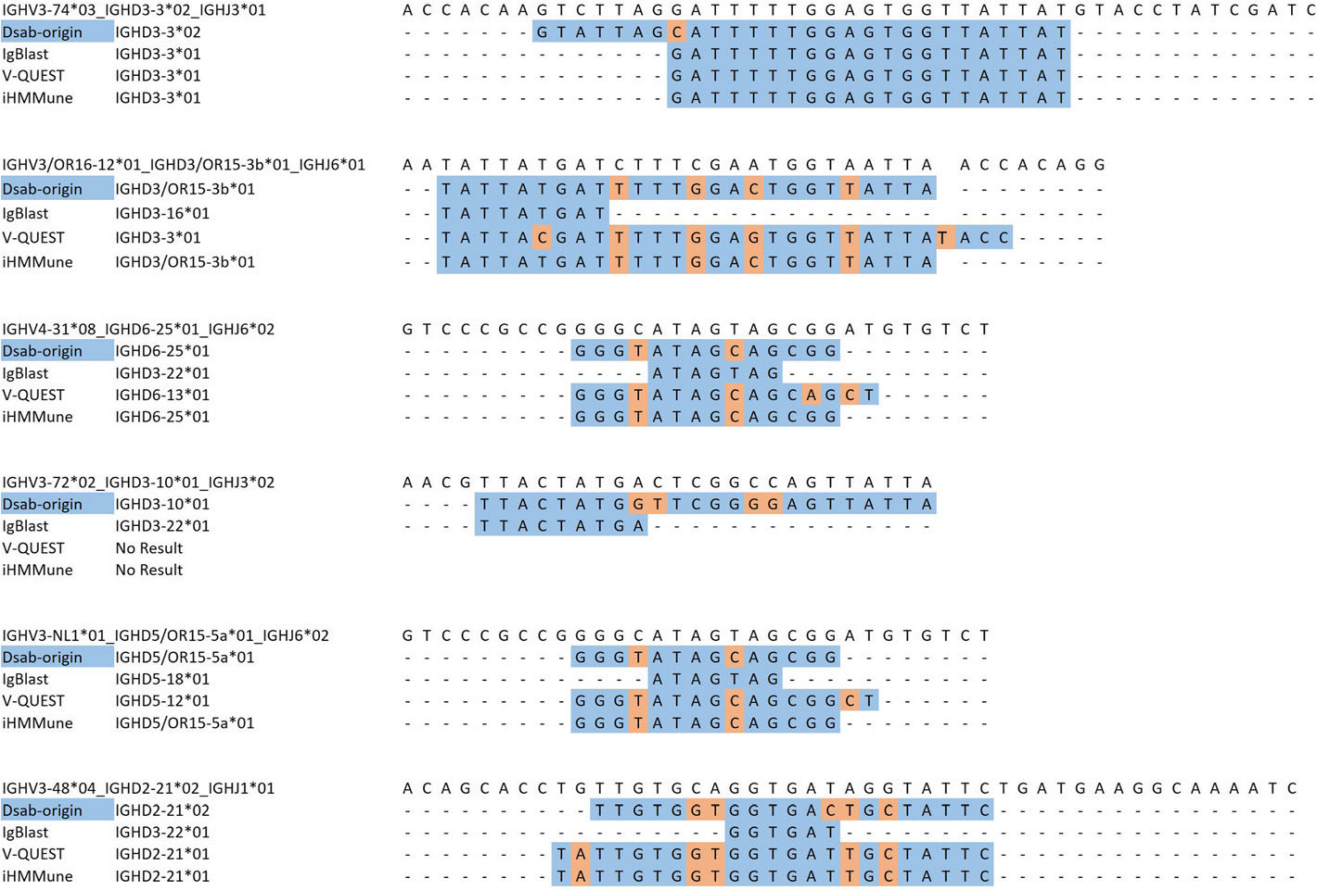
Examples of differentially predicted sequences between DSab-origin and other methods. The blue background represents the mapped sites in the aligned sequences, while the pink background represents the unmapped sites in the aligned sequences

Since it is difficult to obtain experimental data with confident VDJ gene segments usage, except the monoclonal antibody sequencing data, we also chose mutated sequences (40) in Frost’s work [16], which were generated by a simulation program from the human germline IGHV (*n* = 282), IGHD (*n* = 44) and IGHJ (*n* = 13) sequences. The mutated sequences (40) represented about 10% nucleotide divergences from baseline that coincided with the real mutability [32]. With 10,000 simulated sequences, DSab-origin achieved the most accurate prediction in D gene segment. In addition, DSab-origin gave the best performance in holistic prediction of VDJ segments assignment evaluated by weighted rank aggregation (Table 2) in the comparison with other methods (IgBLAST [10], IgSCUEAL [16], IMGT/V-Quest [11], Vdjalign [14], iHMMune [13], Clonanalyst [30], vdj [29], SoDa [33]). Further, sequences with various confident VDJ gene recombination were picked from the mutated sequences (40) as examples of differentially predicted sequences between DSab-origin and other three commonly used methods. In these examples, DSab-origin gave the correct predictions, while other methods were not or got no results (Fig. 1).

**Table 2:** Comparative method performance on mutated sequences (40) simulated data [16]

Methods	IGHV (%)	IGHD (%)	IGHJ (%)
DSab-origin	94.27	62.89	93.51
IgBLAST [10]	96.05	55.64	94.47
IgSCUEAL [16]	99.57	46.95	98.73
IMGT/V-Quest [11]	96.30	53.87	93.38
Vdjalign [14]	83.01	61.48	92.64
iHMMune [13]	90.90	57.70	92.51
Clonanalyst [30]	77.13	58.34	89.20
vdj [29]	75.96	57.35	89.39
SoDa [33]	91.33	54.95	82.82

### Application of DSab-origin on antibody response after influenza vaccination

#### Comparison of immune repertoires before and after vaccination

With the DSab-origin method mentioned above, we then applied it to the TIV vaccination time-series dataset [19]. Firstly, we analyzed the family usage. The assignment of naive B cells represented the gene family usage before TIV vaccination, while the assignments of ASCs and ABCs represented the B cell response after that. It can be seen from Additional file 3: Figure S2 that, IGHV3 took up a large proportion in all donors both in ASCs and ABCs, and IGHV6 and IGHV7 were rarely detected. But, other IGHV family usages showed differences. For instance, the number of IGHV1 gene usage in ASCs and ABCs was less than that in naive B cells in two of three donors, while dnr8 was opposite. The usage of IGHD gene family appeared disorderly and unsystematic that IGHD1~6 were used in all of three cell types with different levels.

**Fig. 2:**
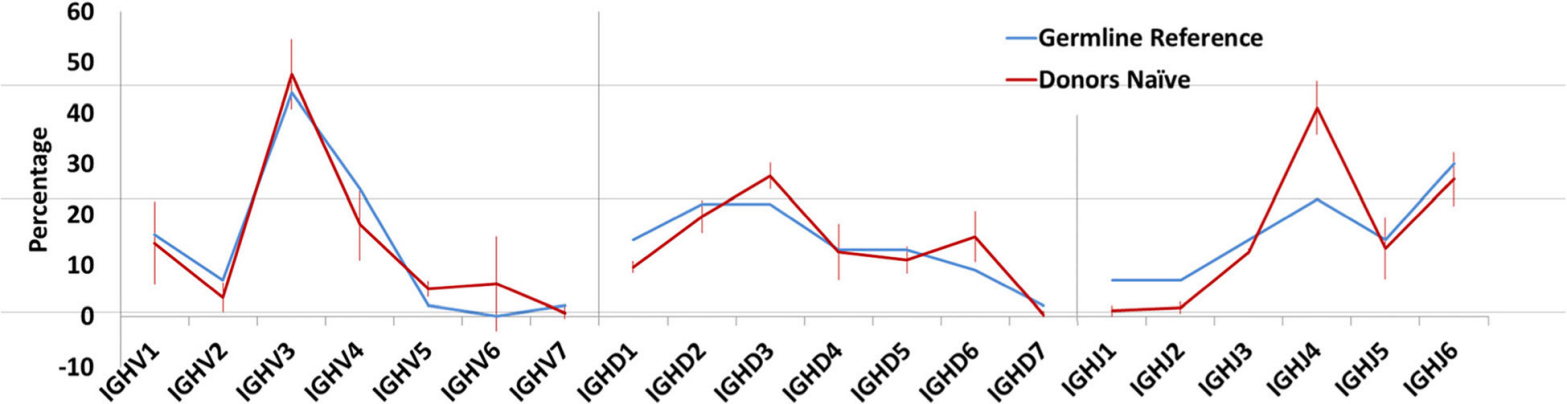
Gene family usage in naive B cells and germline reference. Blue line represents percentage of gene family usage in germline reference, while red line with error bars represents that in three donors

We further analyzed the usages frequencies of VDJ gene family focusing on naive B cells. The usages of naive B cells were similar among the donors, and the average proportions of VDJ gene count that used in each family of three donors were compared with that in the germline references. These two sets of proportions had a Pearson correlation of 0.97, 0.85, 0.85 separately in IGHV family, IGHD family and IGHJ family (Fig. 2). We analyzed the fold changes of gene count used in each family before and after vaccination. Comparing ASCs to the naive B cells, they had distinct changes of family usage frequencies within three donors after vaccination (Additional file 4: Figure S3).

#### IGHV3–7 and IGHD4–17 usage shared by donors after influenza vaccination

**Fig. 3:**
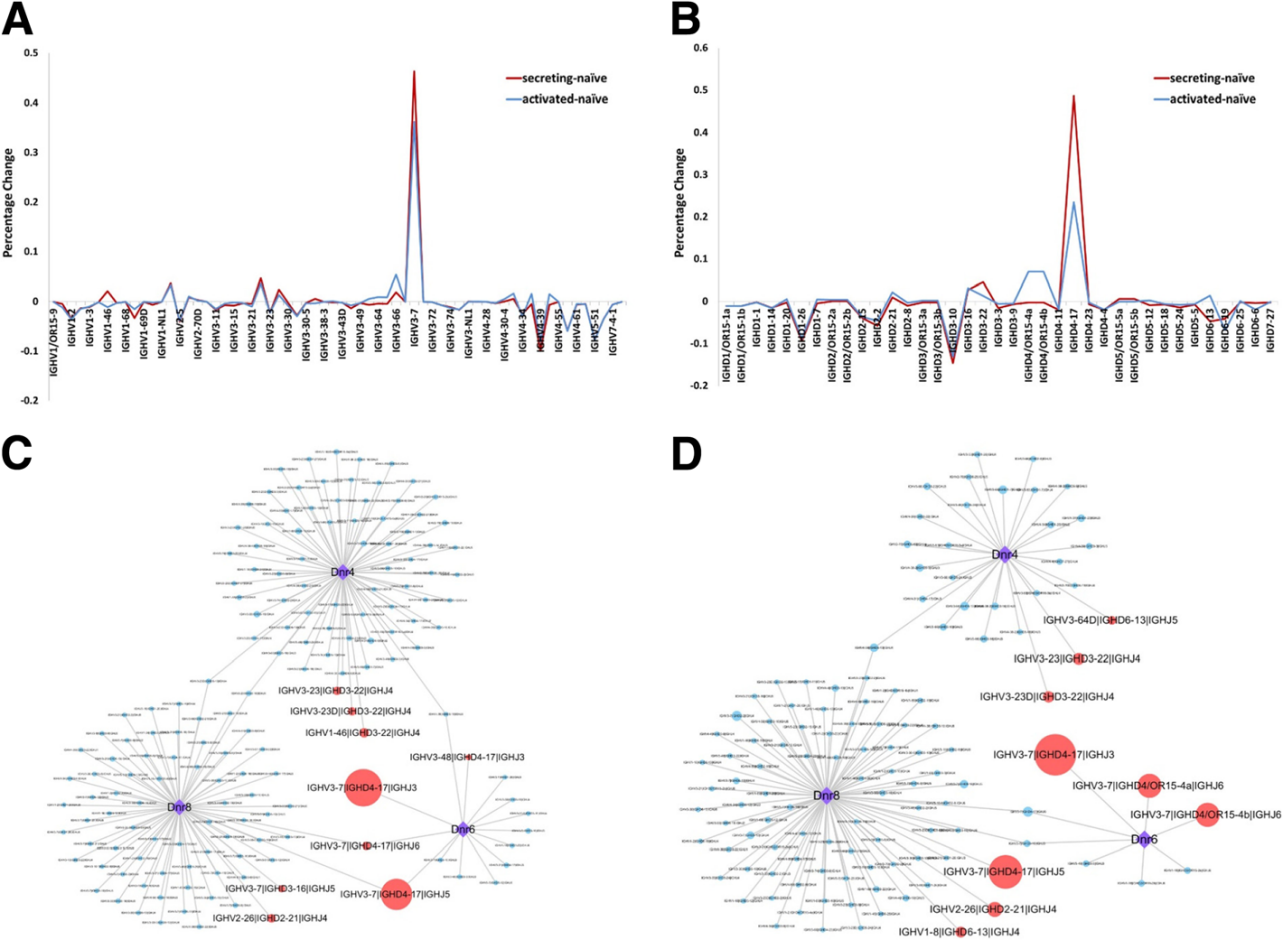
Characters of B cell repertoire before and after influenza vaccination. **a** Percentage changes of IGHV gene usage. Red line represents the changes in ASCs after vaccination with naive B cells as background, while the blue line represents the changes in ABCs under the same condition. **b** Percentage changes of IGHD gene usage. Red line represents the changes in ASCs after vaccination with naive B cells as background. Blue line represents the changes in ABCs. **c** VDJ gene recombination usage in ASCs among three donors. The purple diamonds represent each donor, and points represent each VDJ gene recombination. The size of points represents the expression level changes in ASCs with naive B cells as background. Red points represent the combinations with top 3 expression level in each donor, while others are blue. The lines represent that which donor the combination belongs to. **d** VDJ gene recombination usage in ABCs among three donors. The purple diamonds represent each donor, and points represent each VDJ gene recombination. The size of points represents the expression level changes in ABCs with naive B cells as background. Red points represent the combinations with top 3 expression level in each donor, while others are blue. The lines represent that which donor the combination belongs to

To be more specific, IGHV and IGHD gene usages were investigated individually in naive B cells, ASCs and ABCs. Before TIV vaccination, IGHD gene usage was abundant and various in naive B cells. Then the percentage changes of gene usage were calculated in ASCs and ABCs, where naive B cells were employed as background. IGHV3–7 usage had a significant increase after vaccination in both ASCs and ABCs, while other IGHV gene usages were comparable to the usages before vaccination or decreased. Meanwhile, the result showed that gene usages were consistent in ASCs and ABCs (Fig. 3a). Remarkably, IGHD4–17 had a huge increasing in expression level comparing ASCs and ABCs against naive B cells. There were also small peaks with IGHD3–22 in ASCs and IGHD4\OR15-4a and IGHD4\OR15-4b in ABCs (Fig. 3b). Further, IGHD4–17 was also detected in the top five of usages among IGHD genes in MBCs at day28. Compared to hemagglutinin (HA)-specific MBCs at day28, IGHD4–17 was absent in the top five from MBCs IGHD gene usage at day0 or day90, which contained all the memory B cells in human peripheral blood.

Next, the VDJ gene recombination usages of ASCs and ABCs were calculated as that of naive B cells. For ASCs, a majority of VDJ gene recombination were unique within donors, while the rest of them were shared by no more than two donors. But, the characters could still be detected that IGHV3–7 and IGHD4–17 dominated gene recombination in donor6 and donor8. Although VDJ gene segments usages were disperse in donor4, IGHV3–23 and IGHD3–22 could still stand out from the crowd. In addition, IGHJ3~6 was all used in shared VDJ gene segments combination without specificity (Fig. 3c).

On the other side, ABCs had similar VDJ gene usages with ASCs that most of VDJ gene combinations were occupied by only one donor. But, there still were some shared combinations which basically as same as that in ASCs. Notably, IGHV3–7 and IGHD4–17 also had a high expression level in ABCs, and IGHV3–23 and IGHD3–22 were main combination in donor4 (Fig. 3d).

## Discussion

### Dsab-origin has a high sensitivity in IGHD prediction with best VDJ holistic prediction

In this paper, we developed an IGHD sensitive immune gene assignment method called DSab-origin. The main idea of this method is to conquer them separately focusing on the NDN block, which constitutes most of the CDR3 and contains diversity D and palindromic and non-templated nucleotide additions adjacent to D gene segments, after dividing the query into several blocks. Among D gene segments, sequences are similar within each gene type, but there are not among different gene types (Additional file 5: Figure S4 and Additional file 6: Figure S5). So, it is difficult to predict due to the high mutability of D gene segments and imprecise nucleotide junctions. Since it is important for antibody to contact directly with antigen, and the recombination is usually extremely variable and diverse, we employed a modified k-mers algorithm to maximize the tolerate mismatch. Also, mutable preferences of antibody sequence, such as hot/cold spots [32], were taken into consideration.

Based on above, we used four datasets, which contained simulation data, real experimental data, human monoclonal data and mouse monoclonal antibody sequencing data, to evaluate the performance of DSab-origin. The 57 and 99 unique sequences datasets are real experimental monoclonal data with certain VDJ combination. These datasets with true repertoires can be used to evaluate the performance of DSab-origin on unique sequence. But there is no mixed sequences data with certain different VDJ combinations. For above reason, we employed simulated dataset to compare with other algorithms as the references. The simulated sequences (40) represented about 10% nucleotide divergences from baseline that coincided with the real mutability [16], which may simulate the true repertoires. Meanwhile, S22 Stanford datasets with true and unknown repertoires were also used. To conquer that there was an absent of certain VDJ combination as reference, we analyzed the agreement of predictions with other five algorithms. Although these has no mixed sequences data with certain different VDJ combinations, above datasets gave a comprehensive evaluation on DSab-origin. The performance on 57 and 99 unique sequences datasets indicated that DSab-origin has an advantage in IGHD gene assignment. Mouse monoclonal antibody sequencing data was employed, which illustrated that DSab-origin was robust on different species. Meanwhile, DSab-origin predicted with more than 97% correct alleles in S22 Stanford datasets as experimental data, which means DSab-origin was a suitable method in practice. In simulation data, DSab-origin returned the highest accurate prediction in D gene segment, which might be one of the most important parts for antibody and antigen combination. Though DSab-origin performance on V and J gene assignment was little behind some of other methods, it also achieved high degree of accuracy. Importantly, DSab-origin took the leading position in holistic prediction of VDJ segments assignment evaluated by weighted rank aggregation.

More specifically, in the examples of alignments of sequences, DSab-origin tolerated more unmapped sites in the aligned IGHD segment. These characters have advantages in the prediction for IGHD, which has high mutation rate. Besides, DSab-origin preferred long mapped sequences as the prediction choice, while the extending method in traditional alignment algorithms were not. Importantly, DSab-origin had a stable performance and gave correct prediction in some examples, which some other methods gave no result.

### Application of DSab-origin on three donors after influenza vaccination

To give an example for the application of DSab-origin, a TIV vaccination time-series dataset was assigned by DSab-origin. It should note that the dataset is small for a definite conclusion, and more antibody repertoire datasets in the public domain could be analyzed for a comprehensive study of gene usage after influenza vaccination. The result showed the usage of IGHV3–7 and IGHD4–17 increased predominantly, when comparing ASCs and ABCs to naive B cells, suggesting that both of them might be the main choices by three donors to fight against influenza viruses. The result was consistent with Krause’s study [23], in which they explored the antibody usage after influenza vaccination with a 47 years old healthy female donor. However, the IGHJ gene segments were employed casually. Since IGHJ mainly gets involved in framework region formation, and it is less important in antigen recognition than IGHV and IGHD which contribute to most complementarity determining regions. Due to the similar shared combinations in both ABCs, which belong to MBC lineage and ASCs, they also share the similar gene usage strategies. In addition, IGHD4–17 had a high gene expression level in hemagglutinin (HA)-specific MBCs at day28, indicating that the effective VDJ gene recombination of neutralizing antibody would be added into memory B cell storage to against the following invasion after foreign substances infection.

## Conclusions

In summary, we constructed an IGHD sensitive method DSab-origin to improve the VDJ gene assignment of immunoglobulin, especially for D gene segment. It was designed for a high sensitivity and confidence in IGHD prediction, which gave accuracies around 90% in monoclonal antibody data and average 95.8% in mouse data. Besides, DSab-origin gave the best performance in holistic prediction of VDJ segments assignment comparing with other commonly used methods in simulation data. Then, DSab-origin was applied to a TIV vaccination time-series dataset as an application example. The result showed that the proportions of VDJ gene count used in each gene family had a strong consistency with the germline references in naive B cells. IGHV3–7 and IGHD4–17 were likely to be the dominated gene combination using by the three donors against the influenza vaccine.

## Methods

### Materials

TIV vaccination data was obtained from Sequence Read Archive (SRA) (http://www.ncbi.nlm.nih.gov/sra/) with SRA number: SRP075992 [19]. The Illumina heavy chain sequencing datasets of three healthy adults, who were vaccinated by 2014/2015 trivalent and inactivated seasonal influenza vaccines, were downloaded. The B cell repertoires were sequenced based on naive B cells, MBCs, ABCs and ASCs, respectively. The ASCs and ABCs in day7 (response peak time) were chosen to be analyzed against naive B cells in day0. In addition, MBCs in day0 and day90 were taken into consideration for comparing with ABCs in day7, which were classified as memory B cells lineage. PEAR [34] was used to process the raw data, and quality control was implemented by FASTQ Quality Filter in Fastx-toolkit (http://hannonlab.cshl.edu/fastx_toolkit/).

Validation datasets came from four works separately. Mutated sequences (40) was obtained from Frost’s work [16], which simulated datasets by considering insertions, deletions and mutations, with the known rearrangements. S22 Stanford dataset was obtained from Jackson’s work [28], which comprised 13,153 sequences from an individual who was fully genotyped. 57 and 99 unique sequences were obtained from Zheng’s work [26], which were generated from tonsillar IgG class-switched B cell. Mouse immunoglobulin heavy chain sequencing data was obtained from Yeap’s work [27], which was derived from the sequencing of productive preassembled VDJ allele encoding the immunoglobulin heavy chain in mouse.

### DSab-origin algorithm

Query is artificially divided into three parts: V block (variable V), NDN block (diversity D and additions), and J block (joining J). The algorithm starts with BLAST searches to identify the germline V and J gene hits in V block and J block with the human IGHV and IGHJ germline repertoires obtained from IMGT [25]. Search parameters are set as expected cut-off: 20; word size: 9; mismatch penalty: − 1 in V block search, and expect cut-off: 1000; word size: 7; mismatch penalty: − 3 in J block search, which are consistence with the parameters set by igBlast [10]. Other parameters are set as default.

Then, V block and J block are cut off from query with NDN block remained basing on V and J gene hits. After that, NDN block is processed by modified k-mers algorithm considering the mutable preference of antibody sequence. Firstly, NDN block are split into k length segments and consequently mapped to D germline genes in IGHD germline repertoires. The scores are returned with each D germline genes, as follow:

$$ \mathrm{Score}\kern0.5em =\sum \limits_{\mathrm{i}=0}^{\mathrm{n}}\sum \limits_{\mathrm{j}=0}^{\mathrm{m}}\mathrm{HC}\times \left(\mathrm{K}\hbox{-} \mathrm{Mismatch}\right) $$

i represents the number of segments; n represents the total number of segments; j represents the number of mismatches in each segment mapping; m represents the total number of mismatches; K represents the length of segments; and Mismatch represents the maximum mismatch number that can be tolerated in each segment mapping. Since we traversed each hot/cold spot score from 0.1to 0.9 with a step of 0.1 using real experimental data (57 and 99 datasets), the result indicates that there is a higher accuracy with a higher Hotspots score and a lower Coldspots score. And there is not sensitive with slight change (Additional file 7: Table S1). So we artificially defined that HC equals to 0.5 with a normal mismatch, equals to 0.2 with a Coldspot mismatch and equals to 0.8 with a Hotspot mismatch. The ‘Hotspot’ model is based on the observation that sequence mutability occurs preferably at specific DNA motifs (RGYW, WRCY, WAN), while the ‘Coldspot’ model contains the opposite DNA motifs (SYC, GRS) [32]. Finally, the D germline gene with the maximum score is regarded as the hit.

### TIV sequencing data assignment and analyzation

TIV sequencing data was processed by DSab-origin, and all the sequences were assigned at VDJ gene allele level. Sequences were classified as productive or out-of-frame based on whether the V and J segments were in the same frame; all sequences with stop codons were removed. Based on the VDJ assignment, each sequence was divided into V region, D region, J region and addition regions. The length of each region was calculated, and gene expressions were calculated at gene level in each donor. To analyze the VDJ gene family’s relative expression profile in naive B cells, ASCs and ABCs, each cell type of three donors was assigned. Then the gene family usage frequency was calculated, where there were seven V gene families (IGHV1~7), seven D gene families (IGHD1~7) and six J gene families (IGHJ1~6). The proportion of VDJ gene families were calculated as follow:

$$ {\mathrm{P}}_{\mathrm{f}}=\frac{\sum_{\mathrm{gene}}\mathrm{N}}{\sum_{\mathrm{f}\mathrm{amily}}{\sum}_{\mathrm{gene}}\mathrm{N}} $$

P_f_ represents the proportion of family used in each donor; N represents the number of allele used in the specific gene type.

The fold changes in each family between naive B cells and ASCs were calculated as follow:

$$ {\mathrm{F}}_{\mathrm{f}}={\log}_{10}\frac{\sum_{\mathrm{f}}{\mathrm{N}}_{\mathrm{ASC}}}{\sum_{\mathrm{f}}{\mathrm{N}}_{\mathrm{N}\mathrm{BC}}} $$

F_f_ represents the fold changes in each family; N_ASC_ represents the number of allele used in the specific gene type in this family in ASCs; N_NBC_ represents the number of allele used in the specific gene type in this family in naive B cells.

### Optimization for ranking aggregation

To discover a super list that would be simultaneously as close as possible to all the given ordered lists, an optimization function is defined as follows:

$$ \updelta \ast =\arg \min\ \Phi \left(\updelta \right) $$

where

$$ \Phi \left(\updelta \right)=\sum \limits_{\mathrm{i}=1}^{\mathrm{m}}{\upomega}_{\mathrm{i}}\mathrm{d}\left(\updelta, {\mathrm{L}}_{\mathrm{i}}\right) $$

ω_i_ is the importance weight of ordered list L_i_. Parameter d, which is defined by Spearman distances, is the distance between ‘super list’ δ* and L_i_. The goal of the ranking aggregation is to find δ* which would minimize the total distance between the super list and every ordered list. In this study, weighted rank aggregation is used to evaluate the performance in holistic prediction of VDJ segments assignment.

## Additional files

Additional file 1: **Table S2.** Performance of DSab-origin and other five commonly used algorithms on S22 Stanford data. (DOCX 16 kb)

Additional file 2: **Figure S1.** The performance of DSab-origin as somatic hyper-mutation rates increase. (DOCX 73 kb)

Additional file 3: **Figure S2.** VDJ gene family expression profile of naive B cells, ASCs and ABCs. (DOCX 327 kb)

Additional file 4: **Figure S3.** Frequency changes of gene family usage in ASCs comparing to naive B cells. (DOCX 114 kb)

Additional file 5: **Figure S4.** Alignment of IGHD germlines. (DOCX 578 kb)

Additional file 6: **Figure S5.** Unrooted tree of IGHD germlines. (DOCX 255 kb)

Additional file 7: **Table S1.** Traversing hot/cold spots score. (DOCX 24 kb)

## Abbreviations

ABCs: Activated B cells
ASCs: Antibody-secreting B cells
CDR3: Complementarity determining region 3
MBCs: Memory B cells
PBMC: Peripheral blood mononuclear cell
SHM: Somatic hypermutation
TIV: Trivalent influenza vaccine
