# Supplementary material for: DSab-origin: a novel IGHD sensitive VDJ mapping method and its application on antibody response after influenza vaccination
Source: BMC Bioinformatics. 2019 Mar 14;20:137. doi: 10.1186/s12859-019-2715-7 (PMC6417009; doi:10.1186/s12859-019-2715-7)
Supplement: Supplementary file 1 — Table S2. Performance of DSab-origin and other five commonly used algorithms on S22 Stanford data. (DOCX 16 kb) [file 12859_2019_2715_MOESM1_ESM.docx]

**Table S2** Performance of DSab-origin and other five commonly used algorithms on S22 Stanford data

|  | IGHV | IGHD | IGHJ |
| --- | --- | --- | --- |
| DSab-origin | 97.45% | 97.71% | 99.59% |
| Cloanalyst | 94.38% | 97.37% | 99.61% |
| igBLAST | 43.28% | 89.50% | 99.11% |
| VDJ | 67.26% | 97.91% | 100.00% |
| VDJalign | 91.78% | 97.21% | 100.00% |
| IMGT/V-QUEST | 23.07% | 97.17% | 98.73% |
