# Supplementary material for: DSab-origin: a novel IGHD sensitive VDJ mapping method and its application on antibody response after influenza vaccination
Source: BMC Bioinformatics. 2019 Mar 14;20:137. doi: 10.1186/s12859-019-2715-7 (PMC6417009; doi:10.1186/s12859-019-2715-7)
Supplement: Supplementary file 2 — Figure S1. The performance of DSab-origin as somatic hyper-mutation rates increase. (DOCX 73 kb) [file 12859_2019_2715_MOESM2_ESM.docx]

**
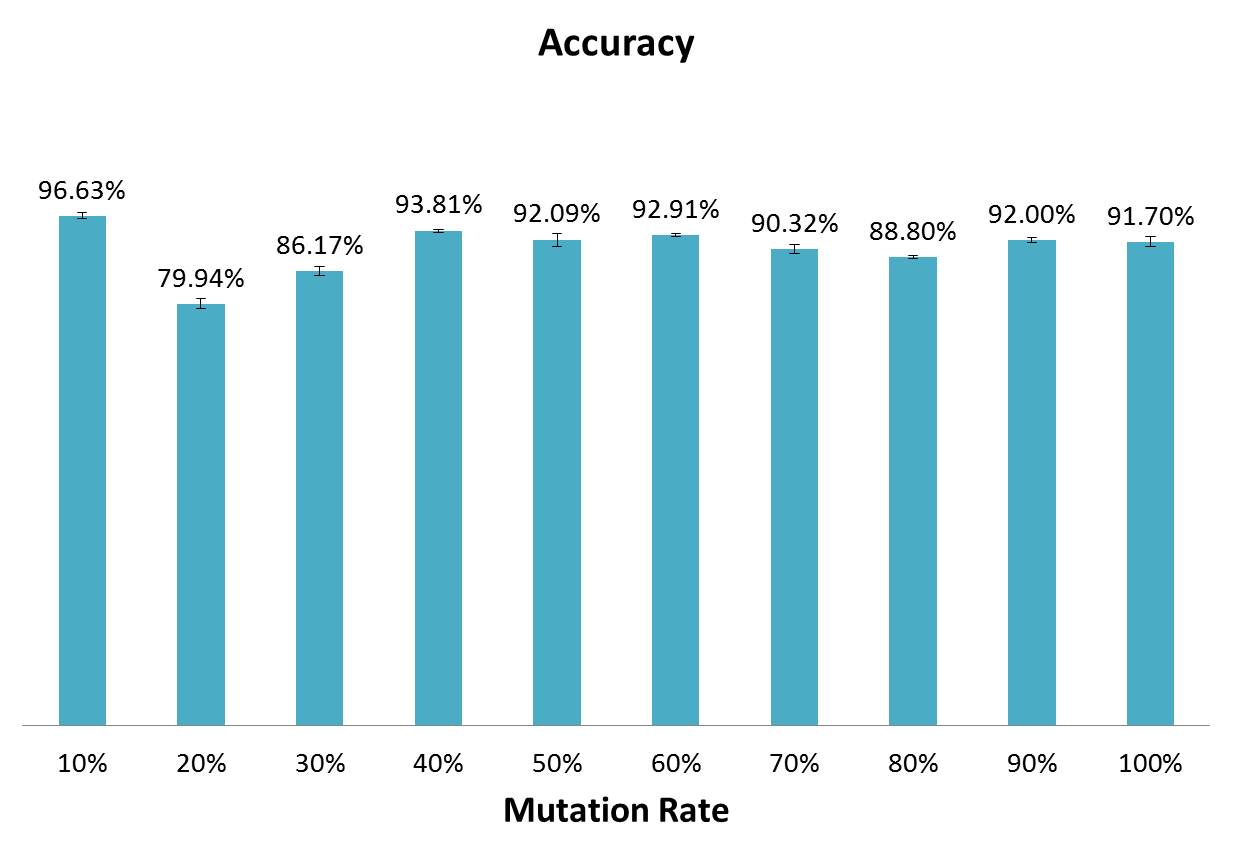
**

**Fig. S1** The performance of DSab-origin as somatic hyper-mutation rates increase. To evaluate the performance of DSab-origin degrade as somatic hyper-mutation rates increase, we generated 10% to 100% mutation rates with a step of 10% using IgSimulator.
