# Supplementary material for: DSab-origin: a novel IGHD sensitive VDJ mapping method and its application on antibody response after influenza vaccination
Source: BMC Bioinformatics. 2019 Mar 14;20:137. doi: 10.1186/s12859-019-2715-7 (PMC6417009; doi:10.1186/s12859-019-2715-7)
Supplement: Supplementary file 3 — Figure S2. VDJ gene family expression profile of naive B cells, ASCs and ABCs. (DOCX 327 kb) [file 12859_2019_2715_MOESM3_ESM.docx]

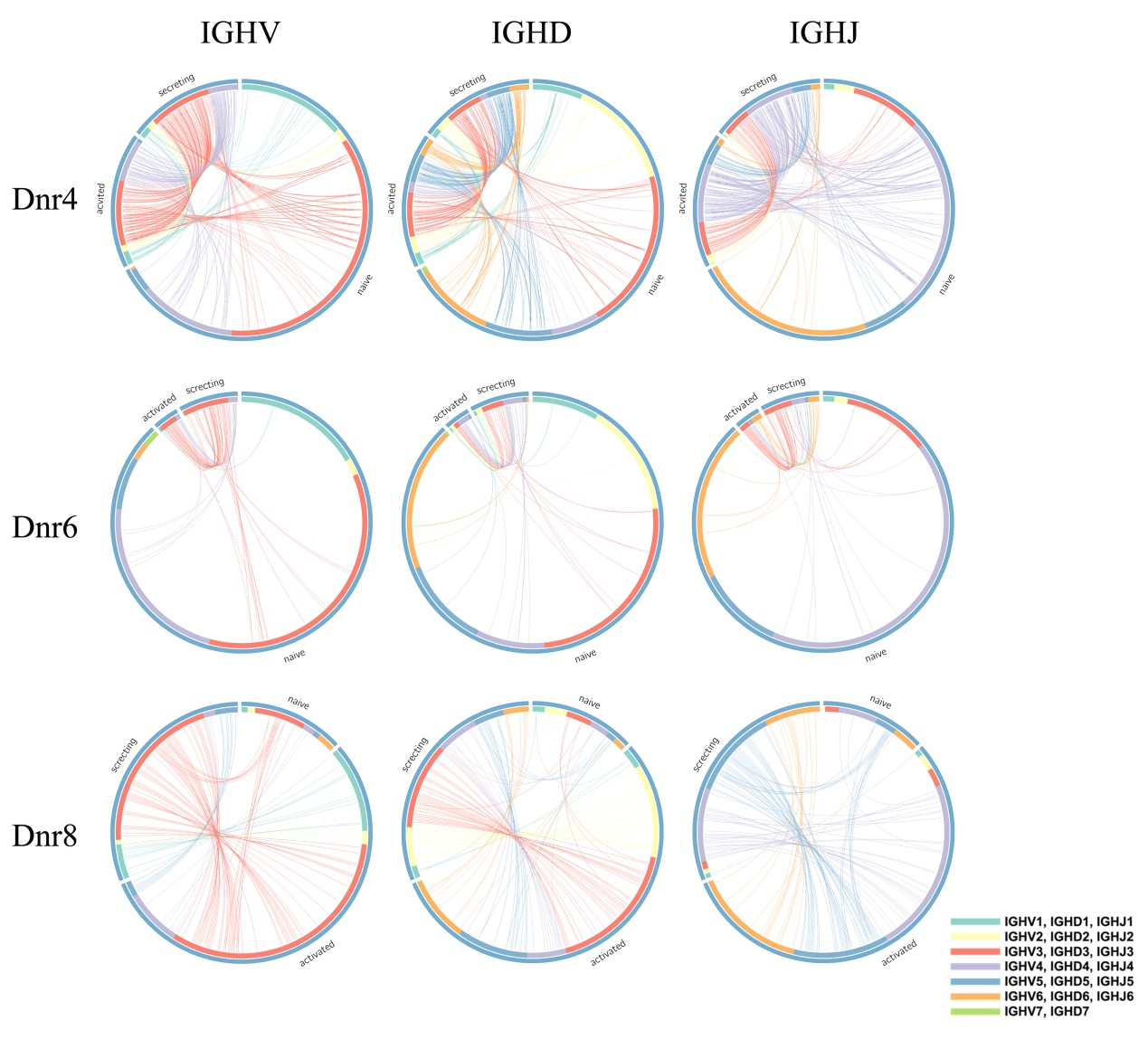


**Fig. S2** VDJ gene family expression profile of naïve B cells, ASCs and ABCs. Each line represents one donor, while each column represents IGHV, IGHD and IGHJ respectively. The outside circle represents the proportion of total number of gene types in naïve B cells, ASCs and ABCs, and the inside circle represents the proportion of the number of gene types within each gene family. The lines connect the same gene type between different cells.
