# Supplementary material for: DSab-origin: a novel IGHD sensitive VDJ mapping method and its application on antibody response after influenza vaccination
Source: BMC Bioinformatics. 2019 Mar 14;20:137. doi: 10.1186/s12859-019-2715-7 (PMC6417009; doi:10.1186/s12859-019-2715-7)
Supplement: Supplementary file 4 — Figure S3. Frequency changes of gene family usage in ASCs comparing to naive B cells. (DOCX 114 kb) [file 12859_2019_2715_MOESM4_ESM.docx]

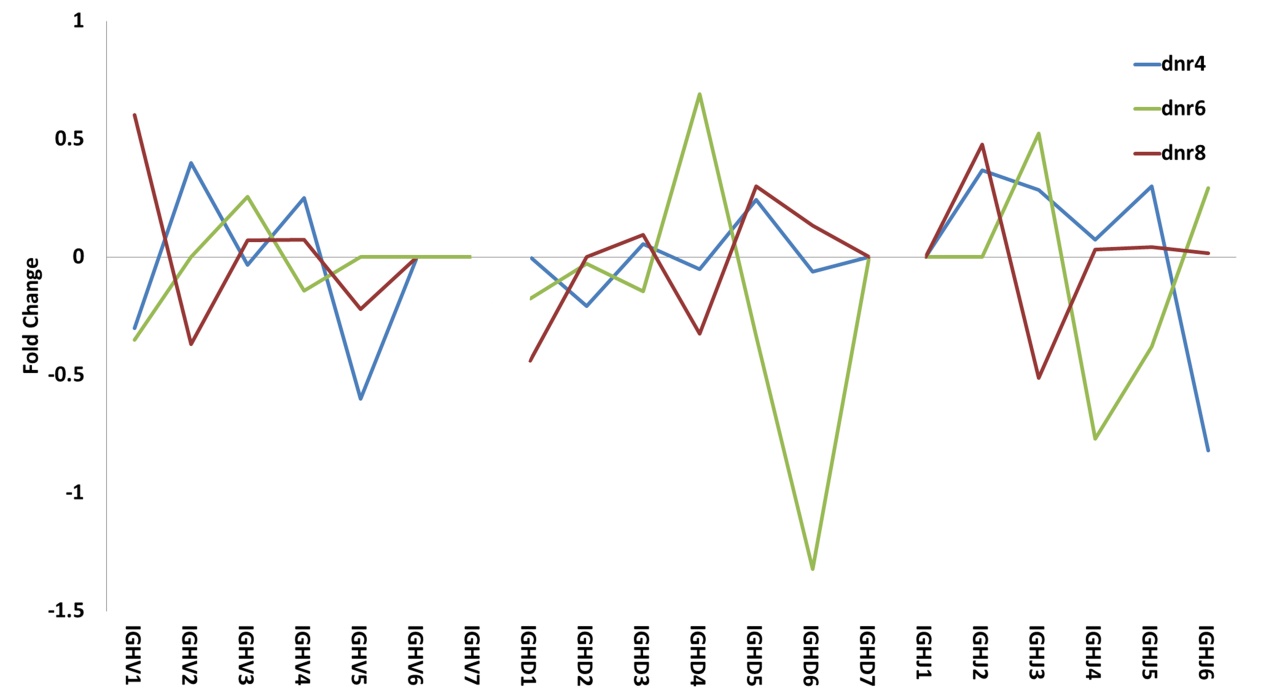


**Fig. S3** Frequency changes of gene family usage in ASCs comparing to naïve B cells. The lines represent the fold changes of the proportion of each VDJ family used in ASCs comparing to naïve B cells.
