# Supplementary figures and images for: DSab-origin: a novel IGHD sensitive VDJ mapping method and its application on antibody response after influenza vaccination
Source: BMC Bioinformatics. 2019 Mar 14;20:137. doi: 10.1186/s12859-019-2715-7 (PMC6417009; doi:10.1186/s12859-019-2715-7)

**
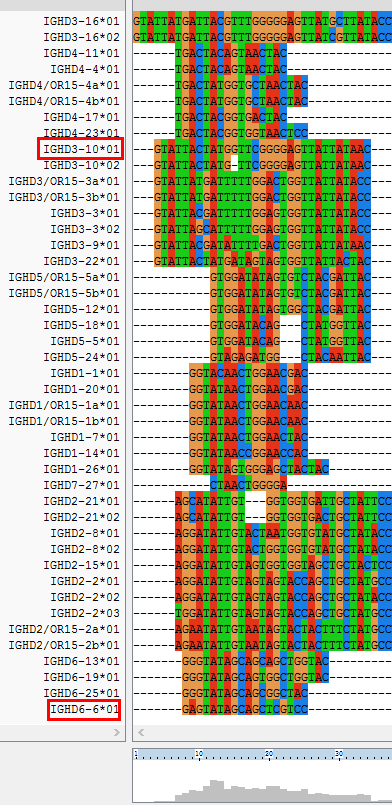
**

**Fig. S4** Alignment of IGHD germlines. IGHD germlines were aligned using clustalX2.

Supplement: Supplementary file 5 — Figure S4. Alignment of IGHD germlines. (DOCX 578 kb) [file 12859_2019_2715_MOESM5_ESM.docx]

**
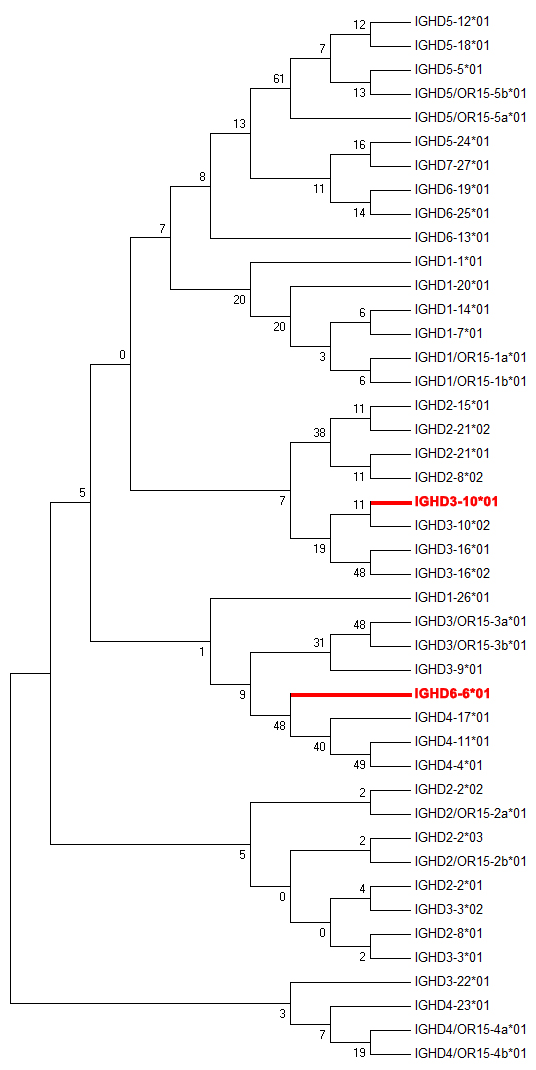
**

**Fig. S5** Unrooted tree of IGHD germlines. Unrooted tree of IGHD germlines was built using MEGA7.0.

Supplement: Supplementary file 6 — Figure S5. Unrooted tree of IGHD germlines. (DOCX 255 kb) [file 12859_2019_2715_MOESM6_ESM.docx]
