# Supplementary material for: DSab-origin: a novel IGHD sensitive VDJ mapping method and its application on antibody response after influenza vaccination
Source: BMC Bioinformatics. 2019 Mar 14;20:137. doi: 10.1186/s12859-019-2715-7 (PMC6417009; doi:10.1186/s12859-019-2715-7)
Supplement: Supplementary file 7 — Table S1. Traversing hot/cold spots score. (DOCX 24 kb) [file 12859_2019_2715_MOESM7_ESM.docx]

**Table S1** Traversing hot/cold spots score

| 57 dataset | | | 99 dataset | | |
| --- | --- | --- | --- | --- | --- |
| hot spot | cold spot | correct prediction | hot spot | cold spot | correct prediction |
| 0.7 | 0.1 | 82.08% | 0.1 | 0.1 | 94.74% |
| 0.7 | 0.2 | 82.08% | 0.1 | 0.2 | 94.74% |
| 0.7 | 0.3 | 82.08% | 0.2 | 0.1 | 94.74% |
| 0.7 | 0.4 | 82.08% | 0.2 | 0.2 | 94.74% |
| 0.8 | 0.1 | 82.08% | 0.2 | 0.3 | 94.74% |
| 0.8 | 0.2 | 82.08% | 0.2 | 0.4 | 94.74% |
| 0.8 | 0.3 | 82.08% | 0.3 | 0.1 | 94.74% |
| 0.8 | 0.4 | 82.08% | 0.3 | 0.2 | 94.74% |
| 0.8 | 0.5 | 82.08% | 0.3 | 0.3 | 94.74% |
| 0.9 | 0.1 | 82.08% | 0.3 | 0.4 | 94.74% |
| 0.9 | 0.2 | 82.08% | 0.4 | 0.1 | 94.74% |
| 0.9 | 0.3 | 82.08% | 0.4 | 0.2 | 94.74% |
| 0.9 | 0.4 | 82.08% | 0.4 | 0.3 | 94.74% |
| 0.9 | 0.5 | 82.08% | 0.4 | 0.4 | 94.74% |
| 0.7 | 0.5 | 81.13% | 0.5 | 0.1 | 94.74% |
| 0.7 | 0.6 | 81.13% | 0.5 | 0.2 | 94.74% |
| 0.8 | 0.6 | 81.13% | 0.5 | 0.3 | 94.74% |
| 0.8 | 0.7 | 81.13% | 0.5 | 0.4 | 94.74% |
| 0.9 | 0.6 | 81.13% | 0.6 | 0.1 | 94.74% |
| 0.9 | 0.7 | 81.13% | 0.6 | 0.2 | 94.74% |
| 0.9 | 0.8 | 81.13% | 0.6 | 0.3 | 94.74% |
| 0.6 | 0.1 | 80.19% | 0.6 | 0.4 | 94.74% |
| 0.6 | 0.2 | 80.19% | 0.7 | 0.1 | 94.74% |
| 0.6 | 0.3 | 80.19% | 0.7 | 0.2 | 94.74% |
| 0.7 | 0.7 | 80.19% | 0.7 | 0.3 | 94.74% |
| 0.7 | 0.8 | 80.19% | 0.7 | 0.4 | 94.74% |
| 0.8 | 0.8 | 80.19% | 0.8 | 0.1 | 94.74% |
| 0.8 | 0.9 | 80.19% | 0.8 | 0.2 | 94.74% |
| 0.9 | 0.9 | 80.19% | 0.8 | 0.3 | 94.74% |
| 0.6 | 0.4 | 79.25% | 0.8 | 0.4 | 94.74% |
| 0.6 | 0.5 | 79.25% | 0.9 | 0.1 | 94.74% |
| 0.4 | 0.1 | 78.30% | 0.9 | 0.2 | 94.74% |
| 0.5 | 0.1 | 78.30% | 0.9 | 0.3 | 94.74% |
| 0.5 | 0.2 | 78.30% | 0.9 | 0.4 | 94.74% |
| 0.5 | 0.3 | 78.30% | 0.2 | 0.5 | 92.98% |
| 0.6 | 0.6 | 78.30% | 0.3 | 0.5 | 92.98% |
| 0.6 | 0.7 | 78.30% | 0.3 | 0.6 | 92.98% |
| 0.5 | 0.4 | 77.36% | 0.3 | 0.7 | 92.98% |
| 0.7 | 0.9 | 77.36% | 0.3 | 0.8 | 92.98% |
| 0.3 | 0.1 | 76.42% | 0.3 | 0.9 | 92.98% |
| 0.4 | 0.2 | 76.42% | 0.4 | 0.5 | 92.98% |
| 0.4 | 0.3 | 75.47% | 0.4 | 0.6 | 92.98% |
| 0.5 | 0.5 | 75.47% | 0.4 | 0.7 | 92.98% |
| 0.3 | 0.2 | 74.53% | 0.4 | 0.8 | 92.98% |
| 0.3 | 0.3 | 74.53% | 0.4 | 0.9 | 92.98% |
| 0.4 | 0.4 | 74.53% | 0.5 | 0.5 | 92.98% |
| 0.5 | 0.6 | 74.53% | 0.5 | 0.6 | 92.98% |
| 0.6 | 0.8 | 73.58% | 0.5 | 0.7 | 92.98% |
| 0.2 | 0.1 | 72.64% | 0.5 | 0.8 | 92.98% |
| 0.3 | 0.4 | 72.64% | 0.5 | 0.9 | 92.98% |
| 0.4 | 0.5 | 72.64% | 0.6 | 0.5 | 92.98% |
| 0.6 | 0.9 | 72.64% | 0.6 | 0.6 | 92.98% |
| 0.2 | 0.2 | 71.70% | 0.6 | 0.7 | 92.98% |
| 0.3 | 0.5 | 71.70% | 0.6 | 0.8 | 92.98% |
| 0.4 | 0.6 | 71.70% | 0.6 | 0.9 | 92.98% |
| 0.4 | 0.7 | 71.70% | 0.7 | 0.5 | 92.98% |
| 0.5 | 0.7 | 71.70% | 0.7 | 0.6 | 92.98% |
| 0.5 | 0.8 | 71.70% | 0.7 | 0.7 | 92.98% |
| 0.5 | 0.9 | 71.70% | 0.7 | 0.8 | 92.98% |
| 0.3 | 0.6 | 70.75% | 0.7 | 0.9 | 92.98% |
| 0.3 | 0.7 | 70.75% | 0.8 | 0.5 | 92.98% |
| 0.3 | 0.8 | 70.75% | 0.8 | 0.6 | 92.98% |
| 0.3 | 0.9 | 70.75% | 0.8 | 0.7 | 92.98% |
| 0.4 | 0.8 | 70.75% | 0.8 | 0.8 | 92.98% |
| 0.4 | 0.9 | 70.75% | 0.8 | 0.9 | 92.98% |
| 0.2 | 0.3 | 68.87% | 0.9 | 0.5 | 92.98% |
| 0.2 | 0.4 | 68.87% | 0.9 | 0.6 | 92.98% |
| 0.2 | 0.5 | 68.87% | 0.9 | 0.7 | 92.98% |
| 0.2 | 0.6 | 68.87% | 0.9 | 0.8 | 92.98% |
| 0.2 | 0.7 | 68.87% | 0.9 | 0.9 | 92.98% |
| 0.2 | 0.8 | 68.87% | 0.1 | 0.3 | 91.23% |
| 0.1 | 0.1 | 66.04% | 0.1 | 0.4 | 91.23% |
| 0.1 | 0.2 | 65.09% | 0.1 | 0.5 | 89.47% |
| 0.1 | 0.3 | 64.15% | 0.1 | 0.6 | 89.47% |
| 0.2 | 0.9 | 60.38% | 0.1 | 0.7 | 89.47% |
| 0.1 | 0.4 | 55.66% | 0.1 | 0.8 | 89.47% |
| 0.1 | 0.5 | 50.00% | 0.1 | 0.9 | 89.47% |
| 0.1 | 0.6 | 50.00% | 0.2 | 0.6 | 89.47% |
| 0.1 | 0.7 | 50.00% | 0.2 | 0.7 | 89.47% |
| 0.1 | 0.8 | 49.06% | 0.2 | 0.8 | 89.47% |
| 0.1 | 0.9 | 49.06% | 0.2 | 0.9 | 89.47% |
